# Supplementary material for: Ambiguous Contribution of Glucocorticosteroids to Acute Neuroinflammation in the Hippocampus of Rat
Source: Int J Mol Sci. 2023 Jul 6;24(13):11147. doi: 10.3390/ijms241311147 (PMC10342621; doi:10.3390/ijms241311147)
Supplement: Supplementary file 1 [file ijms-24-11147-s001.zip › ijms-2461442-supplementary.pdf]

Supplementary material

"Ambiguous contribution of glucocorticosteroids to acute neuroinflammation in the hippocampus of rat" by Liya V. Tret'yakova, Alexey A. Kvichansky, Ekaterina S. Barkovskaya, Anna O. Manolova, Alexey P. Bolshakov, and Natalia V. Gulyaeva

Table S1. Nucleotide sequences of primers used

Gene	Forward primer	Reverse primer
Hprt<br>NM_012583.2	CGT CGT GAT TAG TGA TGATGA AC	CAA GTC TTT CAG TCC TGT CCA TA
Ywhaz<br>NM_013011.4	TTG AGC AGA AGA CGG AAG GT	GAA GCA TTG GGG ATC AAG AA
Il1b<br>NM_031512.2	TCT GTG ACT CGT GGG ATG AT	CAC TTG TTG GCT TAT GTT CTG TC
Il6<br>NM_012589.2	GCC ACT GCC TTC CCT ACT TCA C	GAC AGT GCA TCA TCG CTG TTC ATA C
Tnf<br>NM_012675.3	GTCCAACTCCGGGCTCAGAAT	ACT CCC CCG ATC CAC TCA G
Cx3cl1<br>NM_134455.2	ATC ACC ACC ATC ACC ACC AAC	GAG GAA CAC TTT AAA CCC TCA CAG A
Cx3cr1<br>NM_133534.2	GGA CCT CAC CAT GCC TAC CT	CAC CAA CAG ATT CCC CAC CAG
Ccl2<br>NM_031530.1	GTC GGC TGG AGA ACT ACA AGA G	GGG TCA AGT TCA CAT TCA AAG G
Tgfb1<br>NM_021578.2	GCG CCT GCA GAG ATT CAA GTC AAC	TCA GGC GTA TCA GTG GGG GTC A
Ncf1<br>NM_053734.2	CTG CAG CAA AGG ACA GGA CTG	GGG TCA TGG CCA ACA GGT T

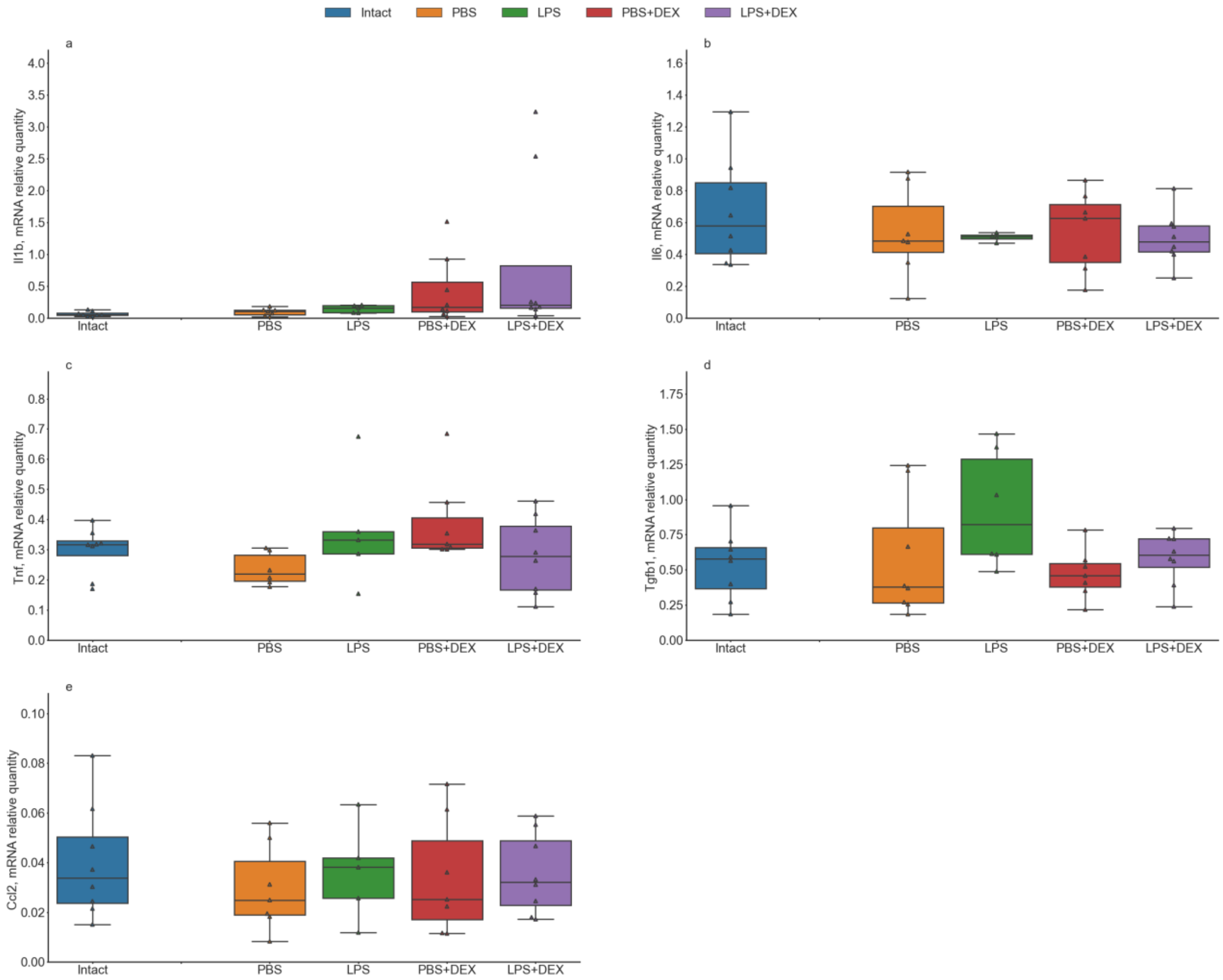

Figure S1. mRNA expression levels of neuroinflammation-associated genes *Il1b* (a), *Il6* (b), *Tnf* (c), *Tgfb1* (d), and *Ccl2* (e) in VH after intrahippocampal injection of PBS, LPS, DEX, or DEX + LPS. n=8.

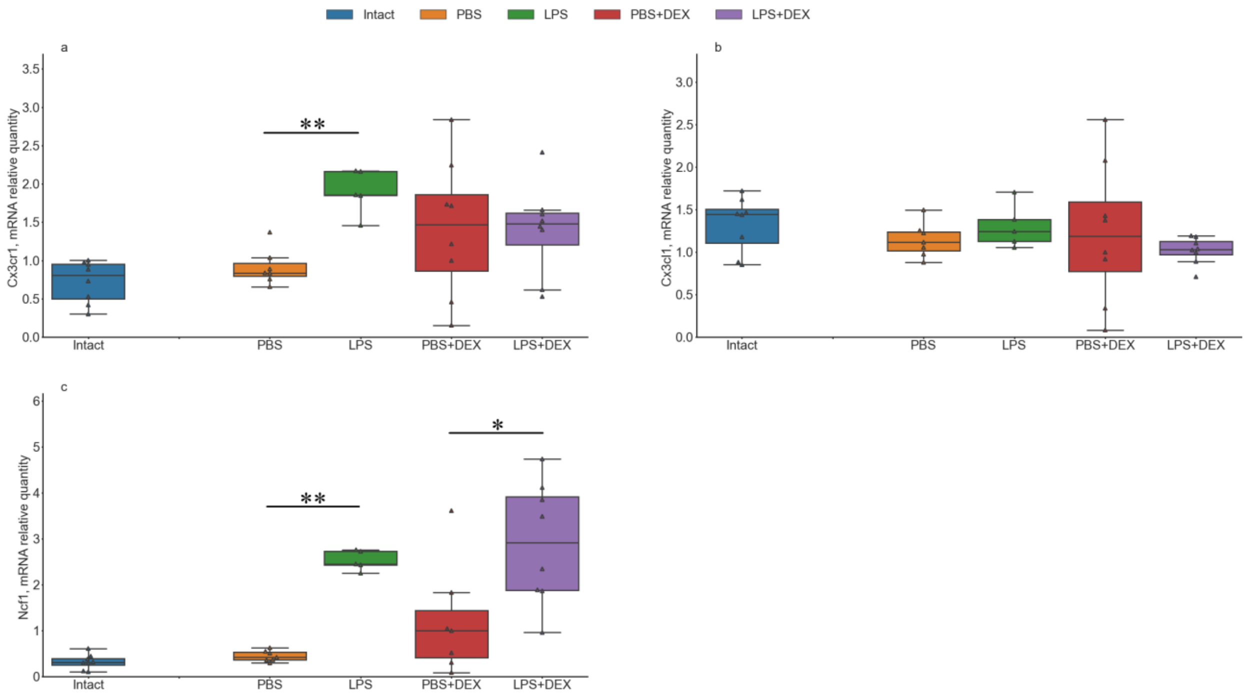

Figure S2. mRNA expression levels of microglial markers genes *Cx3cr1* (a), *Cx3cl1* (b), and *Ncf1* (c) in DH after intrahippocampal injection of PBS, LPS, DEX, or DEX + LPS. n=8. *Cx3cr1*, KW: H=8.0011, p=0.046; *Ncf1*, KW: H=15.4927, p=0.0014. * and **, a trend (0.01≤ p≤0.05) and significant (p<0.01) differences, respectively, according to Mann-Whitney U-test with multiple comparison correction.

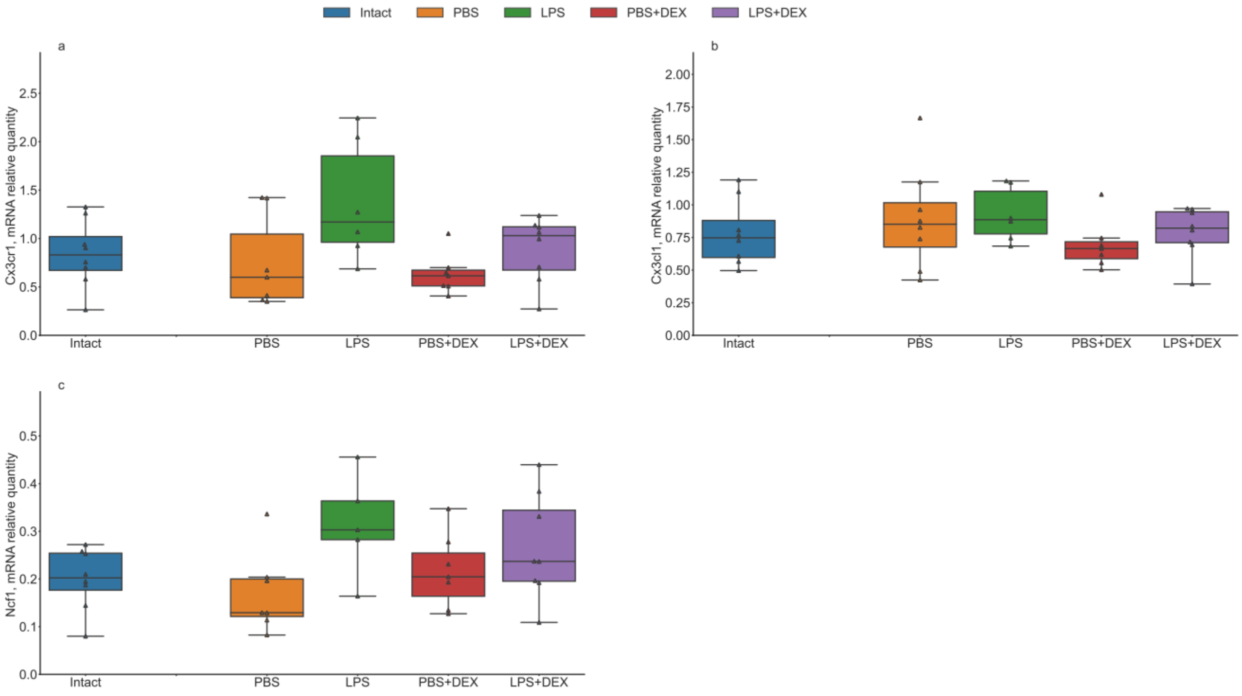

Figure S3. mRNA expression levels of microglial markers genes *Cx3cr1* (a), *Cx3cl1* (b), and *Ncf1* (c) in VH after intrahippocampal injection of PBS, LPS, DEX, or DEX + LPS. n=8.

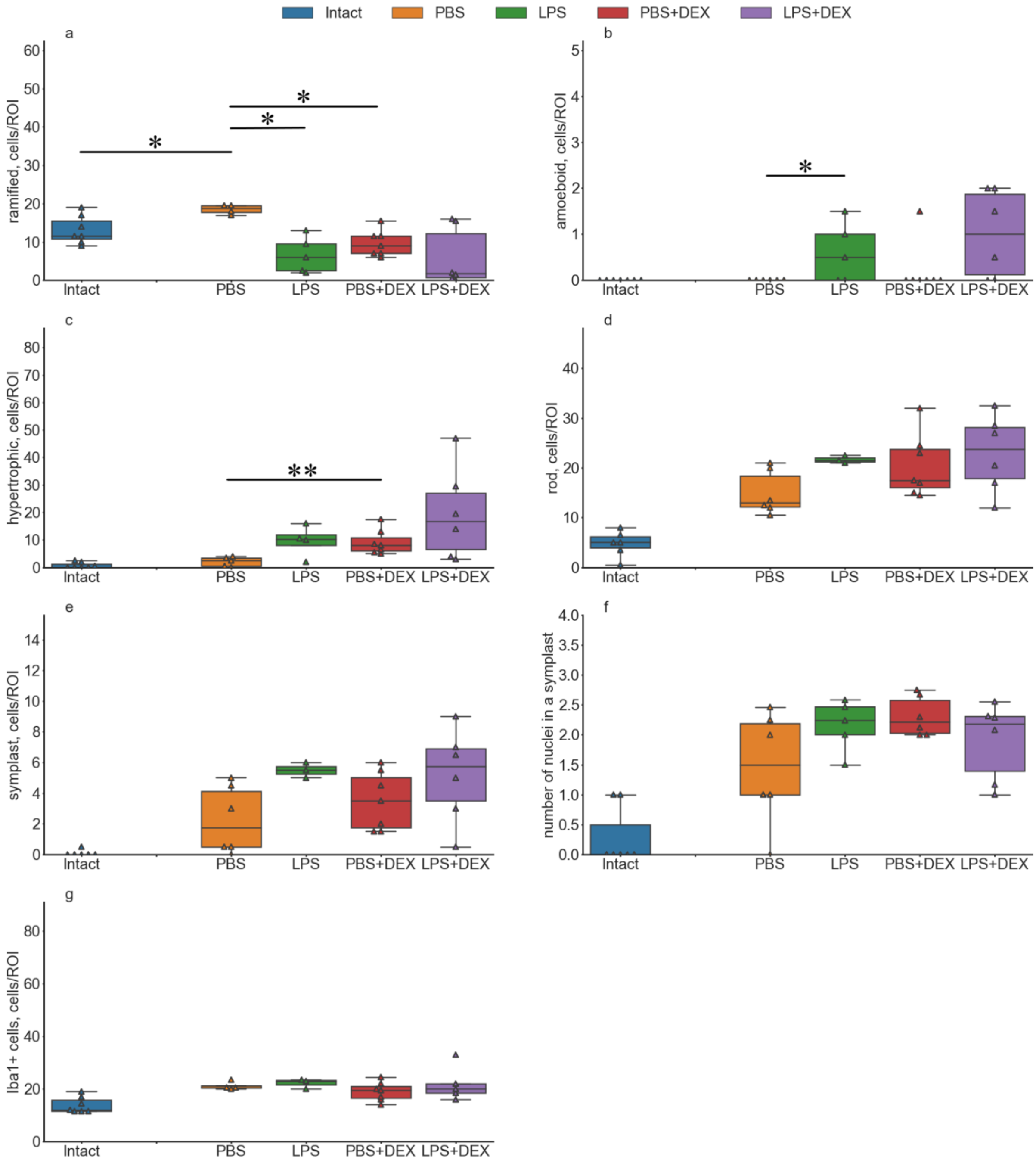

Figure S4. The number of Iba1+ cells of different subtypes in DH after intrahippocampal injection of PBS, LPS, DEX, or DEX + LPS. a, ramified cells, KW: H=10.7388, p=0.0132; b, amoeboid cells, KW: H=8.1273, p=0.0435; c, hypertrophic cells, KW: H=9.0818, p=0.0282; d, rod cells; e, symplasts; f, mean number of nuclei in a symplast; g, number of Iba1+ cells in ROI (region of interest). n=7. * and **, a trend (0.01≤ p≤0.05) and significant (p<0.01) differences, respectively, according to Mann-Whitney U-test with multiple comparison correction.

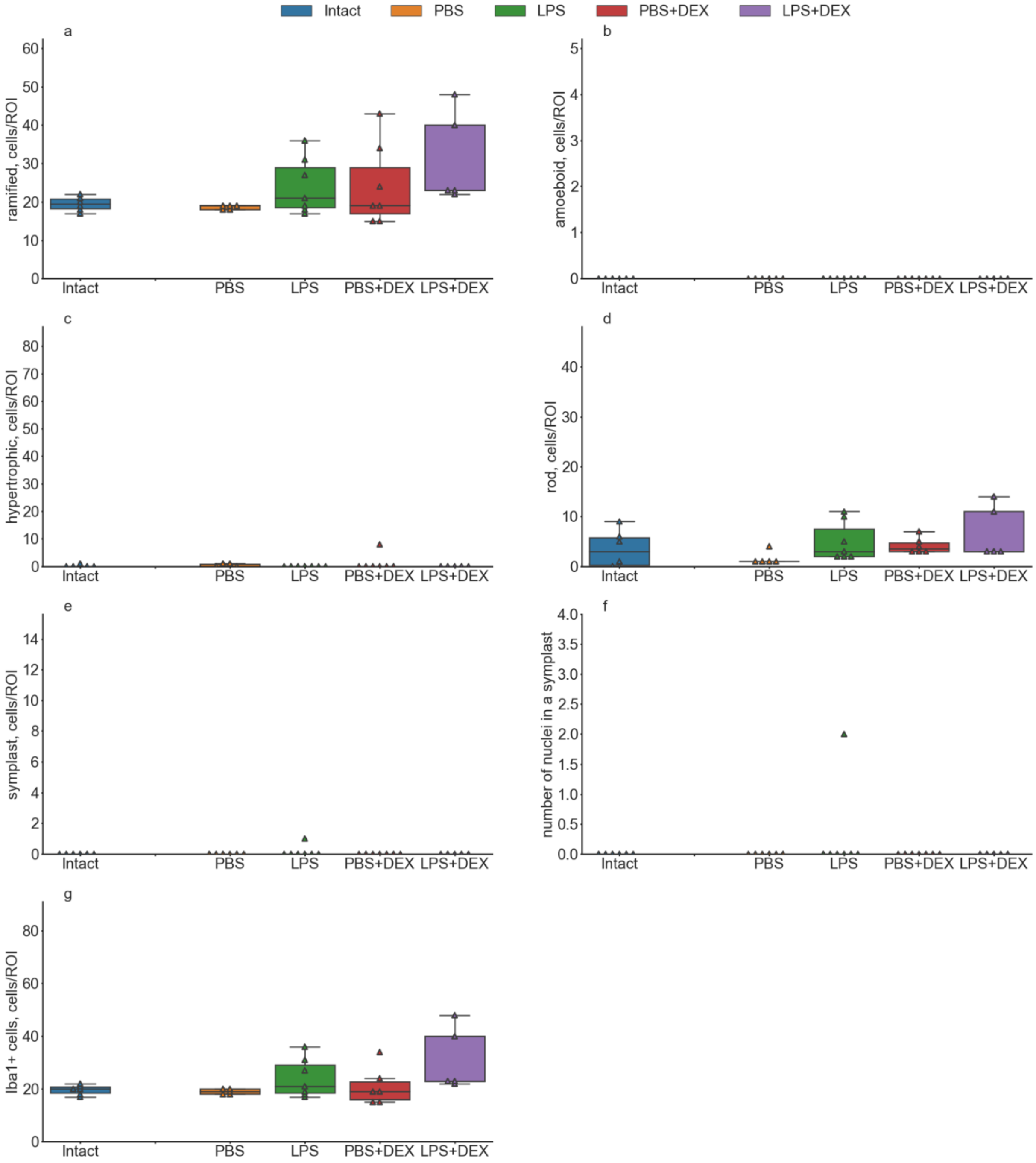

Figure S5. The number of Iba1+ cells of different subtypes in VH after intrahippocampal injection of PBS, LPS, DEX, or DEX + LPS. a, ramified cells; b, amoeboid cells; c, hypertrophic cells; d, rod cells; e symplasts; f, mean number of nuclei in a symplast; g, number of Iba1+ cells in ROI (region of interest). n=7.

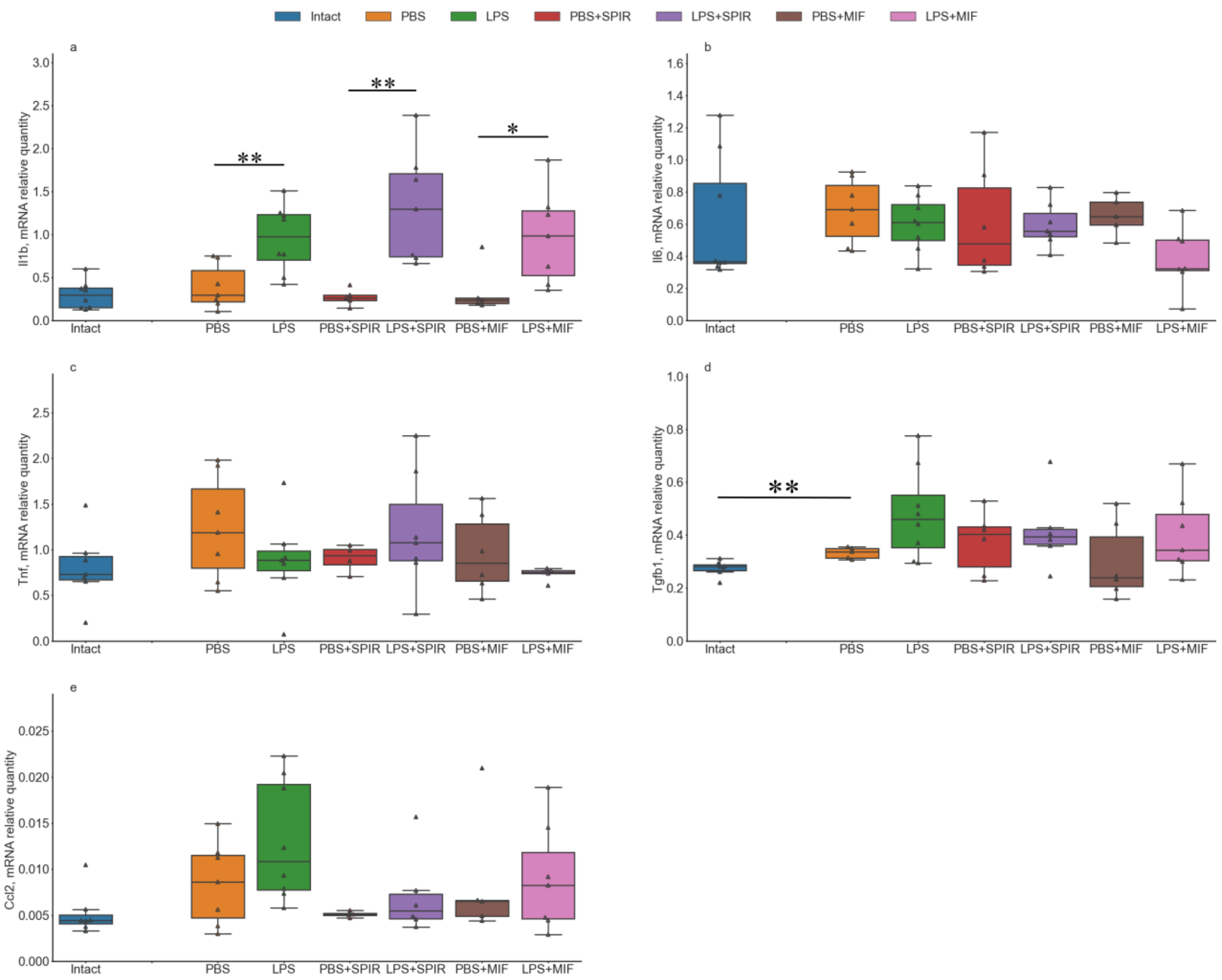

Figure S6. mRNA expression levels of neuroinflammation-associated genes *Il1b* (a), *Il6* (b), *Tnf* (c), *Tgfb1* (d), and *Ccl2* (e) in VH after intrahippocampal injection of PBS, LPS, SPIR, SPIR + LPS, MIF, or MIF + LPS. Intact and LPS groups, n=8; PBS, SPIR + LPS, and MIF + LPS groups, n=7; SPIR and MIF groups, n=6. *Il1b*, KW: H=21.2515, p=0.0007. * and **, a trend (0.00625≤ p≤0.05) and significant (p<0.00625) differences, respectively, according to Mann-Whitney U-test with multiple comparison correction.

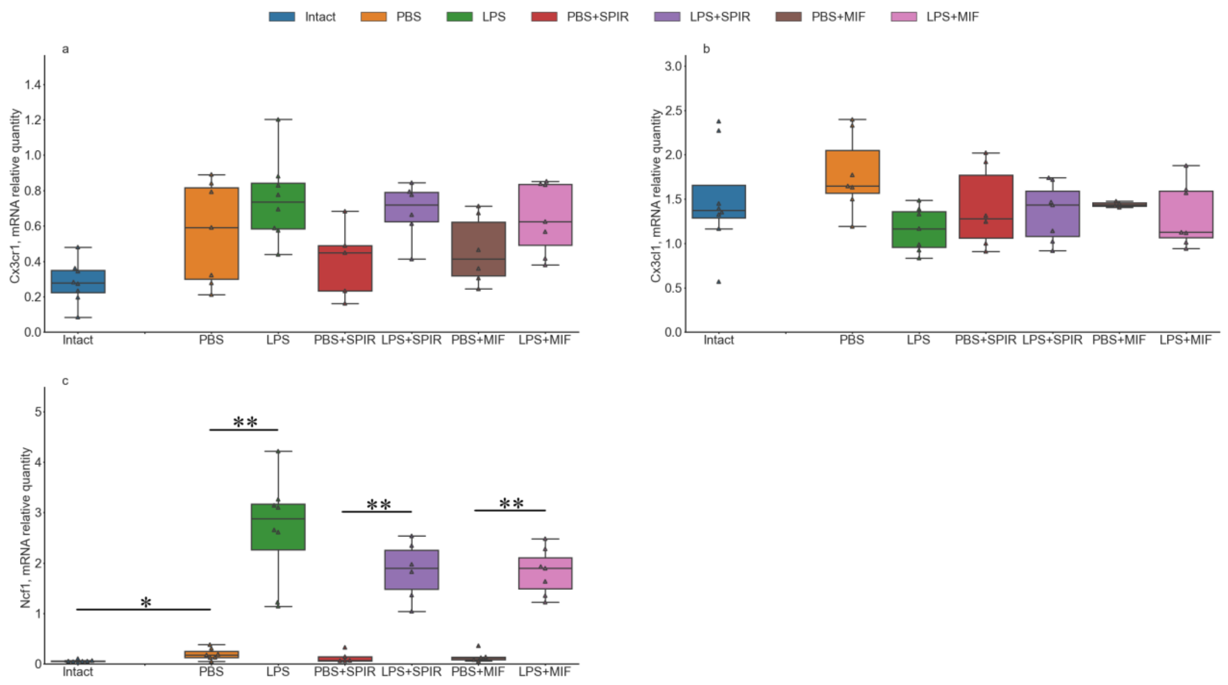

Figure S7. mRNA expression levels of microglial markers genes *Cx3cr1* (a), *Cx3cl1* (b), and *Ncf1* (c) in DH after intrahippocampal injection of PBS, LPS, SPIR, SPIR + LPS, MIF, or MIF + LPS. Intact and LPS groups, n=8; PBS, SPIR + LPS, and MIF + LPS groups, n=7; SPIR and MIF groups, n=6. *Ncf1*, KW: H=28.9908, p=0.00002. * and **, a trend (0.00625≤ p≤0.05) and significant (p<0.00625) differences, respectively, according to Mann-Whitney U-test with multiple comparison correction.

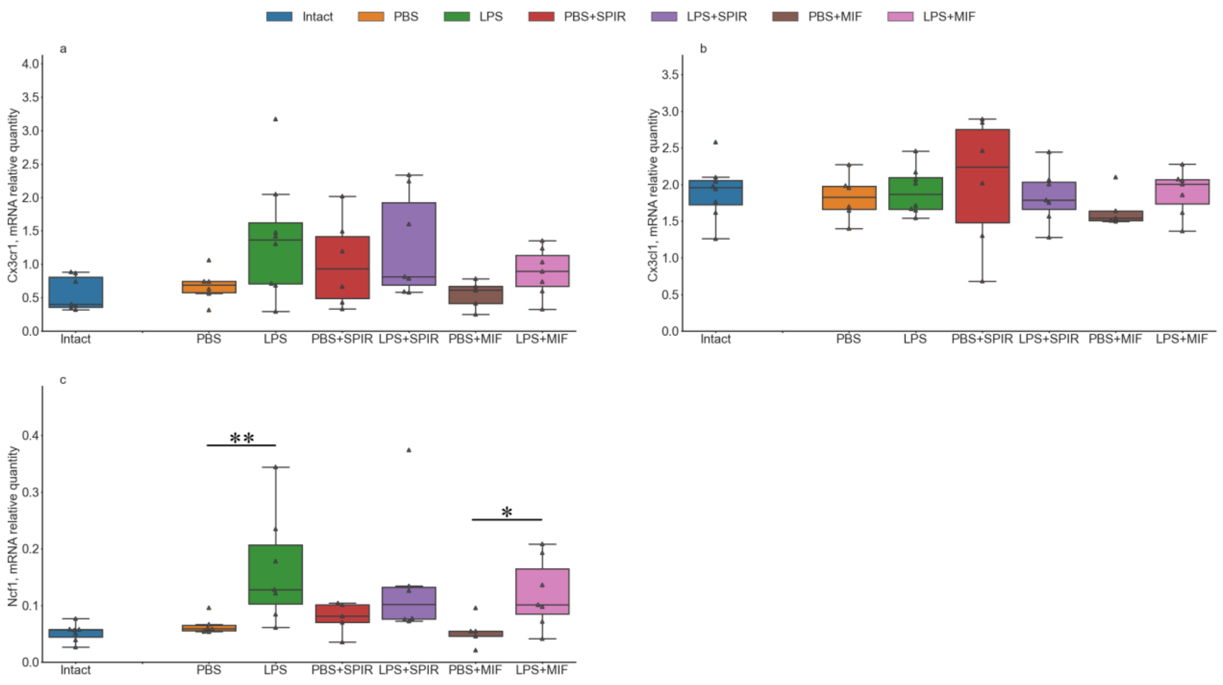

Figure S8. mRNA expression levels of microglial markers genes *Cx3cr1* (a), *Cx3cl1* (b), and *Ncf1* (c) in VH after intrahippocampal injection of PBS, LPS, SPIR, SPIR + LPS, MIF, or MIF + LPS. Intact and LPS groups, n=8; PBS, SPIR + LPS, and MIF + LPS groups, n=7; SPIR and MIF groups, n=6. *Ncf1*, KW: H=14.7948, p=0.0113. * and **, a trend (0.00625≤ p≤0.05) and significant (p<0.00625) differences, respectively, according to Mann-Whitney U-test with multiple comparison correction.

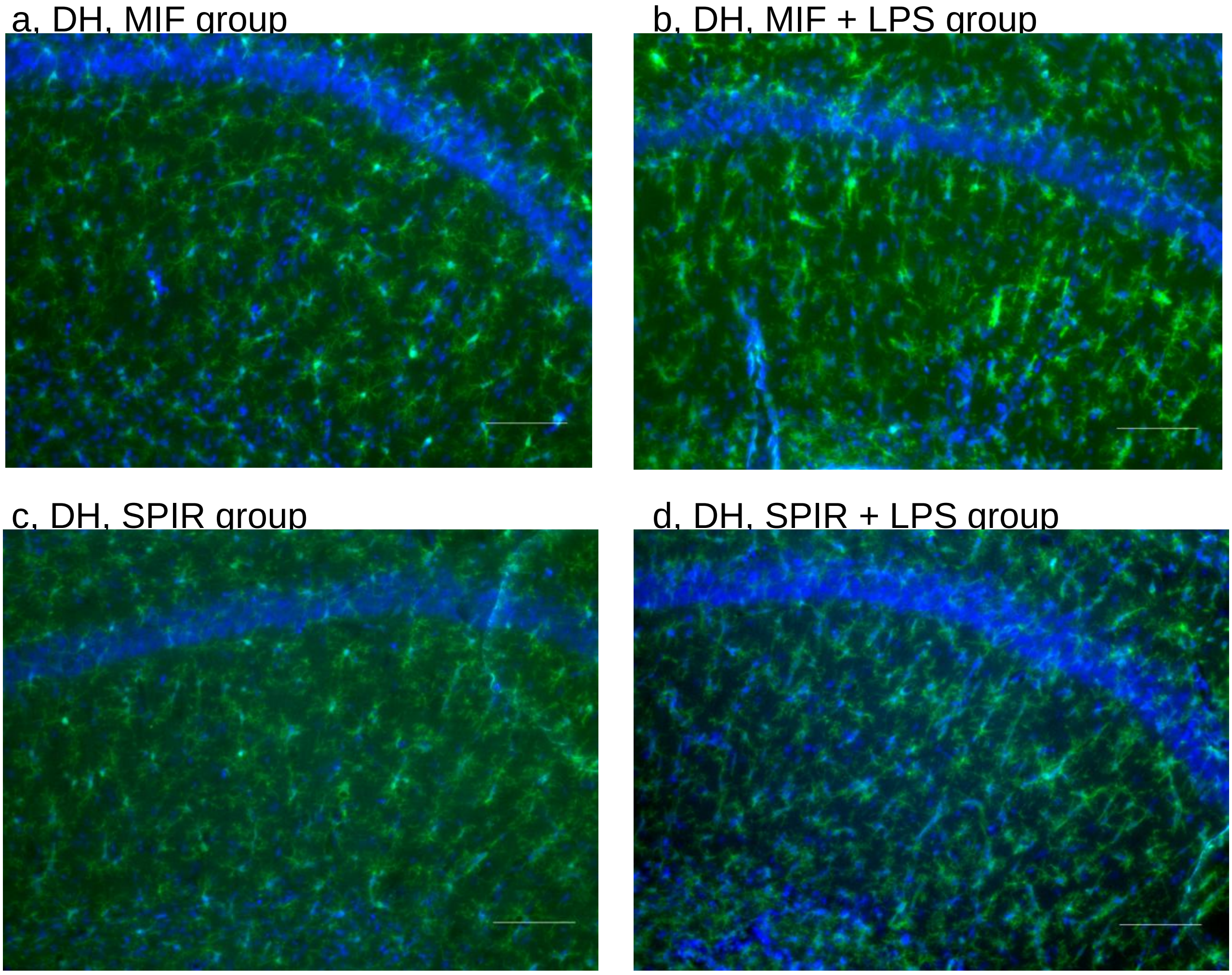

Figure S9. Microglial activation in the DH, representative microphotographs. a, DH MIF group; b, DH MIF + LPS group; c, DH SPIR group; d, DH SPIR + LPS group. Staining with anti-Iba1 and DAPI, x20. Scale bar 100 μm.

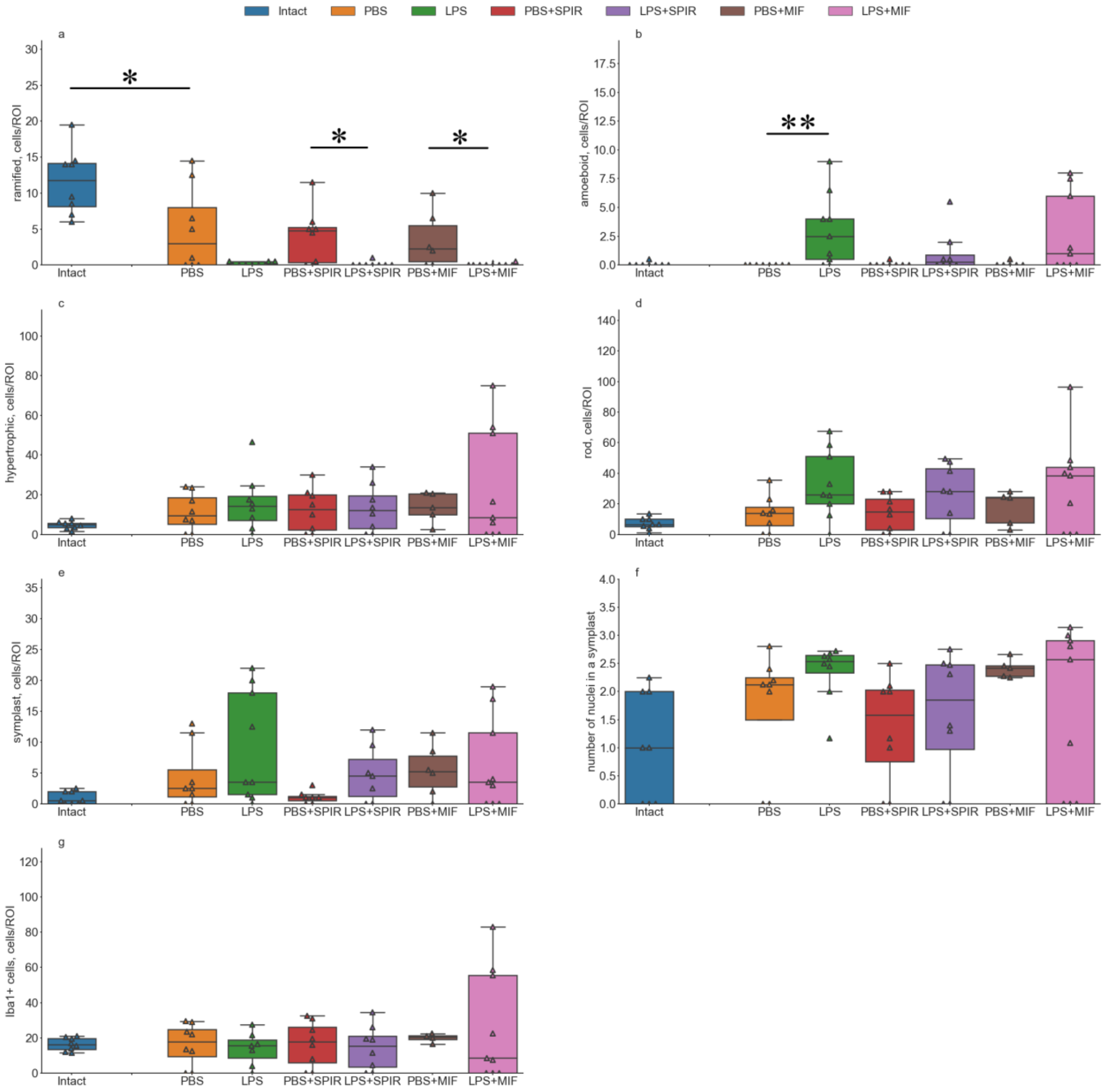

Figure S10. The number of Iba1+ cells of different subtypes in DH after intrahippocampal injection of PBS, LPS, SPIR, SPIR + LPS, MIF, or MIF + LPS. a, ramified cells, KW: H=16.6904, p=0.0051; b, amoeboid cells, KW: H=17.587, p=0.0035; c, hypertrophic cells; d, rod cells; e, symplasts; f, mean number of nuclei in a symplast; g, number of Iba1+ cells in ROI (region of interest). Intact and LPS groups, n=8; PBS, SPIR + LPS, and MIF + LPS groups, n=7; SPIR and MIF groups, n=6. * and **, a trend (0.00625≤ p≤0.05) and significant (p<0.00625) differences, respectively, according to Mann-Whitney U-test with multiple comparison correction.

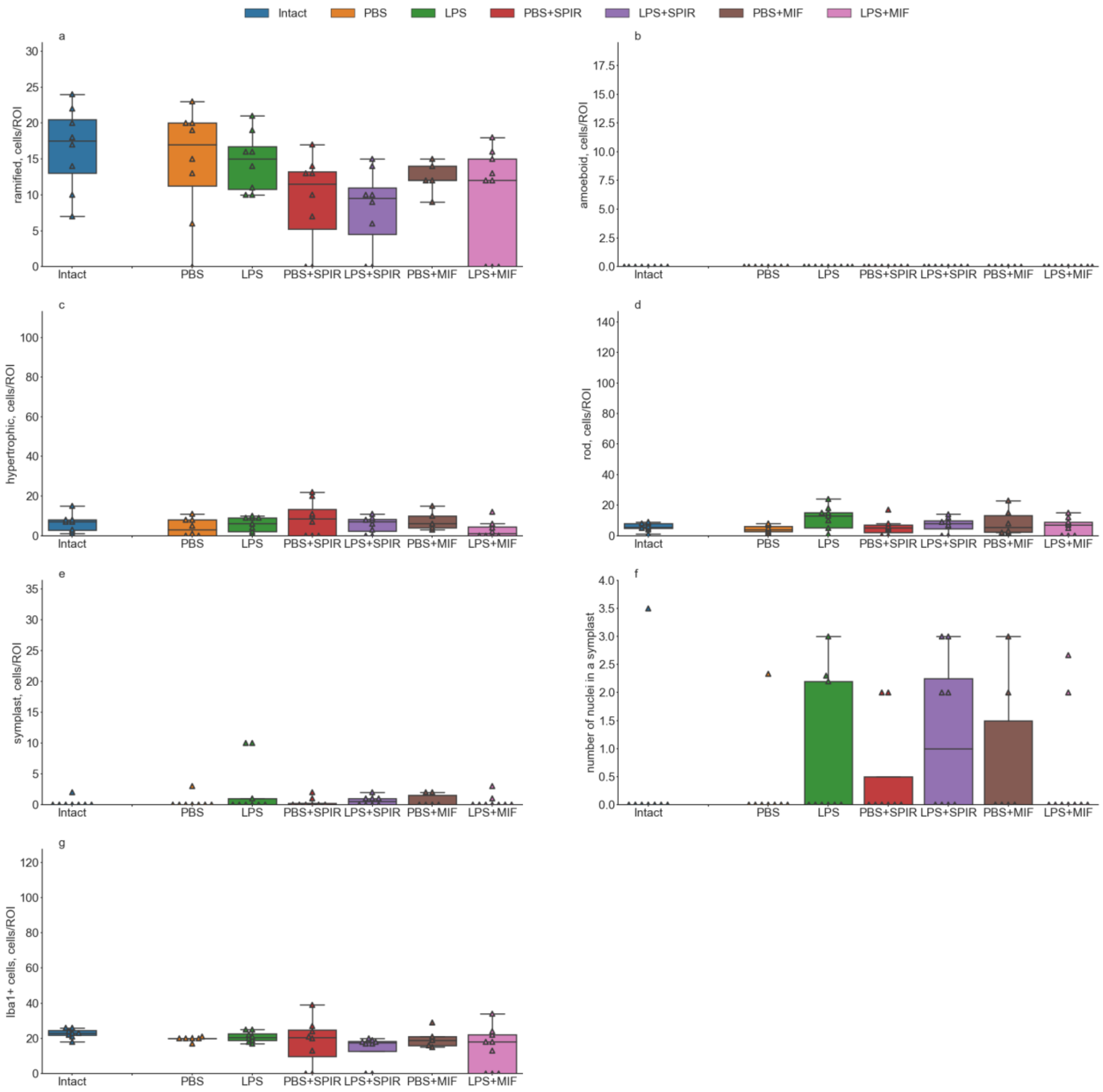

Figure S11. The number of Iba1+ cells of different subtypes in VH after intrahippocampal injection of PBS, LPS, SPIR, SPIR + LPS, MIF, or MIF + LPS. a, ramified cells; b, amoeboid cells; c, hypertrophic cells; d, rod cells; e symplasts; f, mean number of nuclei in a symplast; g, number of Iba1+ cells in ROI (region of interest). Intact and LPS groups, n=8; PBS, SPIR + LPS, and MIF + LPS groups, n=7; SPIR and MIF groups, n=6.

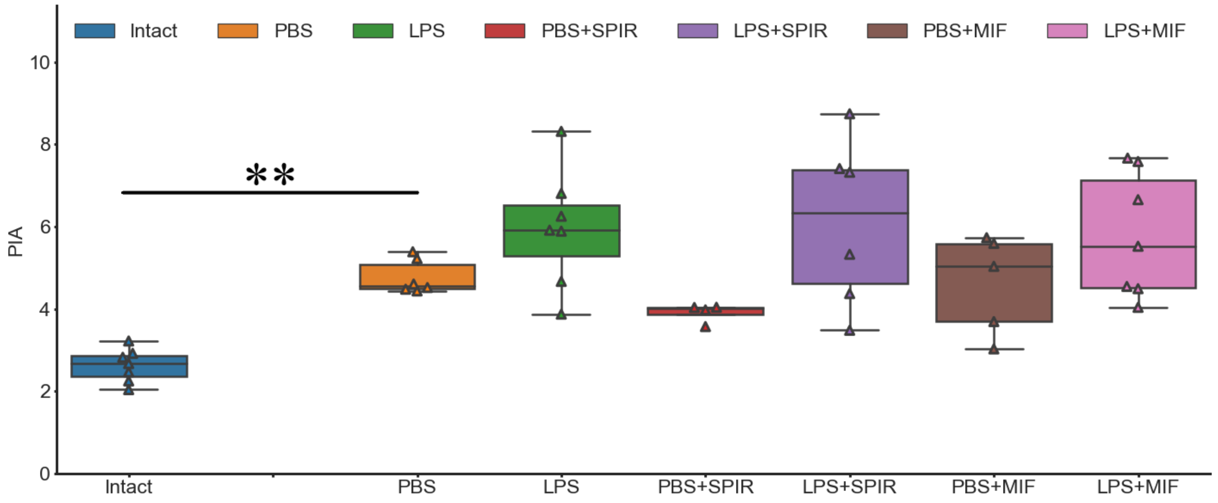

Figure S12. The percentage of Iba1-positively immunostained area (PIA) in DH after intrahippocampal injection of PBS, LPS, SPIR, SPIR+LPS, MIF, or MIF+LPS. Intact and LPS groups, n=8; PBS, SPIR+LPS, and MIF+LPS groups, n=7; SPIR and MIF groups, n=6. **, significant (p<0.00625) differences, respectively, according to Mann-Whitney U-test with multiple comparison correction.

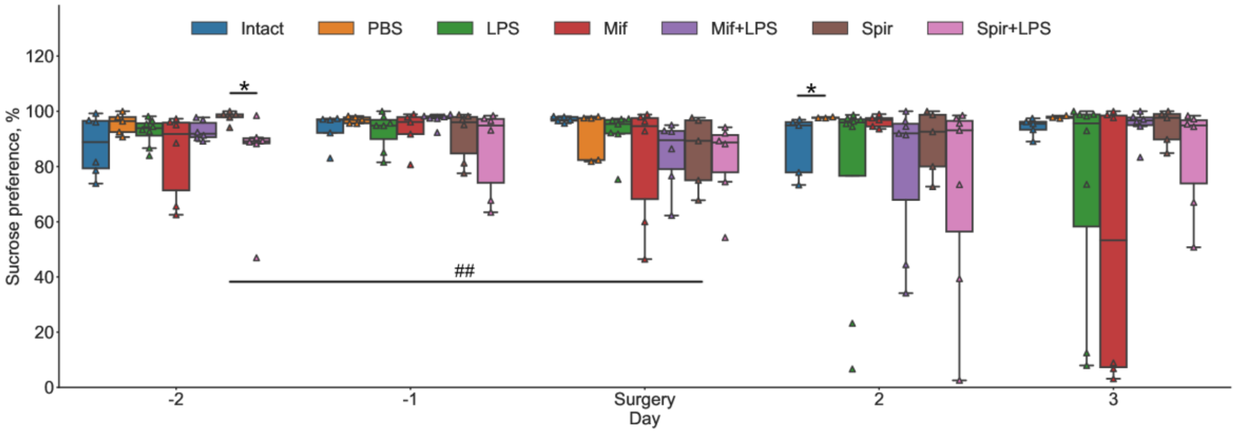

Figure S13. Changes in sucrose consumption of individual animals before and after intrahippocampal injection of PBS, LPS, SPIR, SPIR + LPS, MIF, or MIF + LPS. Intact and LPS groups, n=8; PBS, SPIR + LPS, and MIF + LPS groups, n=7; SPIR and MIF groups, n=6. *, differences at the trend level (0.00625≤p≤0.05) between groups in one day, according to Mann-Whitney U-test with multiple comparison correction. ##, significant (p<0.00625) differences between animals within one group in the certain day, according to the Friedman test with Nemenyi post hoc test.
